# Author Correction: Divergent landscapes of A-to-I editing in postmortem and living human brain

**DOI:** 10.1038/s41467-024-50122-5

**Published:** 2024-07-11

**Authors:** Miguel Rodriguez de los Santos, Brian H. Kopell, Ariela Buxbaum Grice, Gauri Ganesh, Andy Yang, Pardis Amini, Lora E. Liharska, Eric Vornholt, John F. Fullard, Pengfei Dong, Eric Park, Sarah Zipkowitz, Deepak A. Kaji, Ryan C. Thompson, Donjing Liu, You Jeong Park, Esther Cheng, Kimia Ziafat, Emily Moya, Brian Fennessy, Lillian Wilkins, Hannah Silk, Lisa M. Linares, Brendan Sullivan, Vanessa Cohen, Prashant Kota, Claudia Feng, Jessica S. Johnson, Marysia-Kolbe Rieder, Joseph Scarpa, Girish N. Nadkarni, Minghui Wang, Bin Zhang, Pamela Sklar, Noam D. Beckmann, Eric E. Schadt, Panos Roussos, Alexander W. Charney, Michael S. Breen

**Affiliations:** Icahn School of Medicine at Mount Sinai, New York, NY 10029 USA

**Keywords:** Neuroscience, Genomic analysis, Computational biology and bioinformatics, RNA modification

Correction to: *Nature Communications* 10.1038/s41467-024-49268-z, published online 26 June 2024

In this article, the Competing Interests statement was incorrectly given as ‘The authors declare no competing interests.’ And should have been ‘Girish N. Nadkarni is a founder of Renalytix, Pensieve and is a consultant to Renalytix, Heart Test Laboratories and Pensieve. He serves as a scientific advisory board member for Renalytix, Heart Test Laboratories and Pensieve. He also has equity in Renalytix, Pensieve and Verici. He has also consulted for GSK, Reata, Cambridge Capital and GLG consulting. Brian H. Kopell consults and receives consulting fees from Medtronic, Abbott, and Turing Medical. Eric Schadt has equity in, receives a salary for, and is a Corporate Executive Leader for Pathos. He is also a Board of Directors member for Sage Bionetworks and a Board of Directors member for 4YouandMe, a non-profit institute. The original article has been corrected.

