# Supplementary material for: Divergent landscapes of A-to-I editing in postmortem and living human brain
Source: Nat Commun. 2024 Jun 26;15:5366. doi: 10.1038/s41467-024-49268-z (PMC11208617; doi:10.1038/s41467-024-49268-z)
Supplement: Supplementary file 3 — Description of Additional Supplementary Information [file 41467_2024_49268_MOESM3_ESM.pdf]

Description of Additional Supplementary Information

Supplemental Data 1: Summary of Living Brain Project demographics and RNA editing metrics, including external postmortem datasets

Supplemental Data 2: RNA editing summary statistics across the Living Brain Project as well as additional secondary in vitro and in vivo experiments.

Supplemental Data 3: RNA editing summary metrics across secondary postmortem cohorts.

Supplemental Data 4: Cell-specific cataloging of A-to-I sites in postmortem human cortex.

Supplemental Data 5: RNA editing quantitative trait loci in the Living Brain Project.
