# Supplementary material for: Divergent landscapes of A-to-I editing in postmortem and living human brain
Source: Nat Commun. 2024 Jun 26;15:5366. doi: 10.1038/s41467-024-49268-z (PMC11208617; doi:10.1038/s41467-024-49268-z)
Supplement: Supplementary file 9 — Reporting Summary [file 41467_2024_49268_MOESM9_ESM.pdf]

nature portfolio

Corresponding author(s): Michael S. Breen

Last updated by author(s): 05/31/2023

Reporting Summary

Nature Portfolio wishes to improve the reproducibility of the work that we publish. This form provides structure for consistency and transparency in reporting. For further information on Nature Portfolio policies, see our Editorial Policies and the Editorial Policy Checklist.

Statistics

For all statistical analyses, confirm that the following items are present in the figure legend, table legend, main text, or Methods section.

n/a	Confirmed	
☐	☒	The exact sample size (*n*) for each experimental group/condition, given as a discrete number and unit of measurement
☐	☒	A statement on whether measurements were taken from distinct samples or whether the same sample was measured repeatedly
☐	☒	The statistical test(s) used AND whether they are one- or two-sided<br>*Only common tests should be described solely by name; describe more complex techniques in the Methods section.*
☐	☒	A description of all covariates tested
☐	☒	A description of any assumptions or corrections, such as tests of normality and adjustment for multiple comparisons
☐	☒	A full description of the statistical parameters including central tendency (e.g. means) or other basic estimates (e.g. regression coefficient) AND variation (e.g. standard deviation) or associated estimates of uncertainty (e.g. confidence intervals)
☐	☒	For null hypothesis testing, the test statistic (e.g. *F*, *t*, *r*) with confidence intervals, effect sizes, degrees of freedom and *P* value noted<br>*Give P values as exact values whenever suitable.*
☒	☐	For Bayesian analysis, information on the choice of priors and Markov chain Monte Carlo settings
☒	☐	For hierarchical and complex designs, identification of the appropriate level for tests and full reporting of outcomes
☐	☒	Estimates of effect sizes (e.g. Cohen's *d*, Pearson's *r*), indicating how they were calculated

Our web collection on statistics for biologists contains articles on many of the points above.

Software and code

Policy information about availability of computer code

Data collection

Here, we leveraged DLPFC bulk tissue RNA-seq data from a total of 251 living DLPFC samples (comprising 164 biological replicates) together with 233 postmortem DLPFC samples (comprising 233 biological replicates). A subset of 31 living DLPFC tissues were subjected to sn RNA-seq (comprising 22 biological replicates) together with 21 postmortem DLPFC samples (comprising 21 biological replicates). Finally, WGS was used from 155 living and 195 postmortem DLPFC samples with paired bulk tissue RNA-sequencing data.

Data analysis

Bulk RNA sequencing
- STAR (v.2.7.2a) - mapped to the GRCh38
- Picard v2.22.3 - marked duplicate reads and gathered RNA-sequencing short read metrics and distributions (https://github.com/broadinstitute/picard/)
- featureCounts (v1.6.3) - Gene expression analysis and unspecific filtering
- variancePartition R package (v1.20.0) - Normalization of raw counts
-dtangle v2.0.9 - Cellular deconvolution of bulk tissue
-REDItools v2.
-RNAEditingIndexer v.1
-fastQTL v.2.184

single-cell RNA sequencing
- CellRanger software (v7.0) - genome alignment
- SoupX - remove contaminating ambient RNA
- Seurat R package (v4.2.0) - identifiaction of highly variable gene features, cell-type clustering

- AEI method v1.0 - computing Alu editing index in bulk RNA sequencing data and snRNAseq

RNA editing site detection and annotation
-JACUSA2 (https://github.com/dieterich-lab/JACUSA2) - Detection of de-novo sites
-samtools mpileup v1.16 - Detection of known sites
-ANNOVAR (10/24/2019) - annotation of sites
-PHAST package (v3.15) - conservation metrics
Detecting dynamically regulated A-to-I sites
-limma R package (v.3.36.3) - linear modeling

For manuscripts utilizing custom algorithms or software that are central to the research but not yet described in published literature, software must be made available to editors and reviewers. We strongly encourage code deposition in a community repository (e.g. GitHub). See the Nature Portfolio guidelines for submitting code & software for further information.

Data

Policy information about availability of data

All manuscripts must include a data availability statement. This statement should provide the following information, where applicable:

- Accession codes, unique identifiers, or web links for publicly available datasets
- A description of any restrictions on data availability
- For clinical datasets or third party data, please ensure that the statement adheres to our policy

All LBP data generated for this report will be deposited publicly when manuscript is accepted for publication

Research involving human participants, their data, or biological material

Policy information about studies with human participants or human data. See also policy information about sex, gender (identity/presentation), and sexual orientation and race, ethnicity and racism.

Reporting on sex and gender	Female and human pindividuals were considered for this study. Supplemental table 1 and figure 1 describes the distribution of the cohorts.
Reporting on race, ethnicity, or other socially relevant groupings	In the living cohort, 140 individuals were Caucasian and 24 individuals were of mixed races. In the postmortem cohort, 179 individuals were Caucasian and 54 were of mixed race. Supplemental table 1 describes the distribution.
Population characteristics	There are 397 individuals in the living brain project. Supplemental table 1 and Figure 1 describes the population.
Recruitment	Recruitment was performed through large healthcare system in New York City
Ethics oversight	The current study was approved by the Icahn School of Medicine at Mount Sinai's Institutional Review Board

Note that full information on the approval of the study protocol must also be provided in the manuscript.

Field-specific reporting

Please select the one below that is the best fit for your research. If you are not sure, read the appropriate sections before making your selection.

☒ Life sciences ☐ Behavioural & social sciences ☐ Ecological, evolutionary & environmental sciences

For a reference copy of the document with all sections, see nature.com/documents/nr-reporting-summary-flat.pdf

Life sciences study design

All studies must disclose on these points even when the disclosure is negative.

Sample size	Here, we leveraged DLPFC bulk tissue RNA-seq data from a total of 251 living DLPFC samples (comprising 164 biological replicates) together with 233 postmortem DLPFC samples (comprising 233 biological replicates). A subset of 31 living DLPFC tissues were subjected to sn RNA-seq (comprising 22 biological replicates) together with 21 postmortem DLPFC samples (comprising 21 biological replicates). Finally, WGS was used from 155 living and 195 postmortem DLPFC samples with paired bulk tissue RNA-sequencing data.
Data exclusions	We excluded RNA seq samples with fewer than 10000 A-to-I editing sites
Replication	We replicated the initial findings across multiple independent post-mortem data sets
Randomization	N/A
Blinding	N/A

Reporting for specific materials, systems and methods

We require information from authors about some types of materials, experimental systems and methods used in many studies. Here, indicate whether each material, system or method listed is relevant to your study. If you are not sure if a list item applies to your research, read the appropriate section before selecting a response.

Materials & experimental systems

n/a	Involved in the study
☒	☐ Antibodies
☒	☐ Eukaryotic cell lines
☒	☐ Palaeontology and archaeology
☒	☐ Animals and other organisms
☐	☒ Clinical data
☒	☐ Dual use research of concern
☒	☐ Plants

Methods

n/a	Involved in the study
☒	☐ ChIP-seq
☒	☐ Flow cytometry
☒	☐ MRI-based neuroimaging

Clinical data

Policy information about clinical studies

All manuscripts should comply with the ICMJE guidelines for publication of clinical research and a completed CONSORT checklist must be included with all submissions.

Clinical trial registration	N/A
Study protocol	N/A
Data collection	N/A
Outcomes	N/A
